# Multilayered skyscraper microchips fabricated by hybrid “all-in-one” femtosecond laser processing

**DOI:** 10.1038/s41378-019-0056-3

**Published:** 2019-05-06

**Authors:** Chaowei Wang, Liang Yang, Chenchu Zhang, Shenglong Rao, Yulong Wang, Sizhu Wu, Jiawen Li, Yanlei Hu, Dong Wu, Jiaru Chu, Koji Sugioka

**Affiliations:** 1 CAS Key Laboratory of Mechanical Behavior and Design of Materials, Department of Precision Machinery and Precision Instrumentation, University of Science and Technology of China, 230026 Hefei, China; 2 Institute of Industry and Equipment Technology, Hefei University of Technology, 230009 Hefei, China; 3 School of Instrument Science and Opto-electronics Engineering, Hefei University of Technology, 230009 Hefei, China; 4 RIKEN Center for Advanced Photonics, 2-1 Hirosawa, Wako, Saitama, 351-0198 Japan

**Keywords:** Nanofluidics, Optical materials and structures

## Abstract

Multilayered microfluidic channels integrated with functional microcomponents are the general trend of future biochips, which is similar to the history of Si-integrated circuits from the planer to the three-dimensional (3D) configuration, since they offer miniaturization while increasing the integration degree and diversifying the applications in the reaction, catalysis, and cell cultures. In this paper, an optimized hybrid processing technology is proposed to create true multilayered microchips, by which **“**all-in-one” 3D microchips can be fabricated with a successive procedure of 3D glass micromachining by femtosecond-laser-assisted wet etching (FLAE) and the integration of microcomponents into the fabricated microchannels by two-photon polymerization (TPP). To create the multilayered microchannels at different depths in glass substrates (the top layer was embedded at 200 μm below the surface, and the underlying layers were constructed with a 200-μm spacing) with high uniformity and quality, the laser power density (13~16.9 TW/cm^2^) was optimized to fabricate different layers. To simultaneously complete the etching of each layer, which is also important to ensure the high uniformity, the control layers (nonlaser exposed regions) were prepared at the upper ends of the longitudinal channels. Solvents with different dyes were used to verify that each layer was isolated from the others. The high-quality integration was ensured by quantitatively investigating the experimental conditions in TPP, including the prebaking time (18~40 h), laser power density (2.52~3.36 TW/cm^2^) and developing time (0.8~4 h), all of which were optimized for each channel formed at different depths. Finally, the eight-layered microfluidic channels integrated with polymer microstructures were successfully fabricated to demonstrate the unique capability of this hybrid technique.

## Microfluidics: 3D chips to power next-gen experiments

Newly-developed manufacturing technologies enable the construction of powerful microfluidics chips. Just as how silicon computer chips leaped in capability thanks to the development of three-dimensional circuitry, microfluidics chips—which conduct experiments on liquids in micrometer-sized channels—stand to benefit from the same development. Researchers led by Dong Wu, from the University of Science and Technology of China, and Koji Sugioka, from Japan’s RIKEN Center for Advanced Photonics, developed a methodology that leverages femtosecond laser pulses to fabricate multi-layer glass microfluidics channels. The team validated their process by creating an eight-layered glass microfluidics device with layers of channels separated by just 200 µm. 3D microfabrication technologies such as this have the capacity to enable point-of-care diagnostics, the modeling of multiple organ systems, and complex studies involving particle mixing and separation.

## Introduction

In recent years, highly integrated microfluidic chips, which provide portable, eco-friendly, safe, and highly efficient experimental platforms, are highly desired due to their capability in both fundamental science and practical applications to chemical experiments^1^, environmental monitoring^2^, biological assays^3^, tissue engineering^4^, medical diagnosis^5^, etc. Existing techniques, such as photolithography^6^, soft lithography^7^, and nanoimprint lithography^8^ have been well established to fabricate two-dimensional (2D) planar microfluidic chips. Although these microchips are effective in a wide range of applications, only the 2D fabrication capability of these techniques makes it difficult to integrate more complex components for more complicated applications including particle separation^9^, mixing^10^, and point-of-care diagnose^11^. If microfluidic chips with true three-dimensional (3D) configurations can be constructed, they will enable fluids to flow in a 3D environment, which can miniaturize the chip size^12^ and increase the efficiency^13^. Furthermore, the 3D multilayered microfluidic chip, where each layer has different functions, will highly enhance the functionality and performance to diversify the applications^14–18^ similarly to the development of 3D Si integrated circuits. Because of the significant advantages of 3D multilayered microfluidic devices, some attempts have been made to fabricate them with various materials and techniques. For example, a paper-based 3D microfluidic device fabricated by stacking layers of patterned papers with double-sided adhesive tapes enables one to simultaneously test four different samples with up to four different analyses and display the results in a side-by-side configuration^19^. However, it is difficult to control the channel shapes by this method, and the optical transmittance necessary for the optical analysis is not very good due to the paper-based devices. Another 3D microfluidic device was fabricated by stacking polymethyl methacrylate (PMMA) substrates layer by layer, where the photolithography-patterned PMMA substrates were bonded using solvent-assisted thermal compression bonding^20^. However, this method consists of multiple complex operation steps, and it is challenging to achieve a secured bonding between the layers. Some 3D biomimetic microvascular microchips embedded in a hydrogel matrix were fabricated by omnidirectional 3D printing^21^. Although this method can fabricate channels with complex shapes, the mechanical properties and stability of the channels are poor.

Despite many efforts in fabricating 3D microfluidic chips, the progress remains limited because no technology can flexibly and simply integrate different types of functional components in each layer. The difficulties of such integration are mainly attributed to two aspects. First, the inner wall of the microchannel fabricated by the conventional planar processes is not flat^22^, which requires very high precision in the integration. Second and more importantly, the conventional methods can only process 2D microstructures, and it remains challenging to integrate 3D functional microstructures in microfluidic channels^23^. Consequently, a flexible and tunable processing technique, which is compatible for both microchip preparation and functional microstructure integration, is urgently required. In recent years, femtosecond laser micromachining (FLM) has shown a special capability of prototyping 3D micro/nanostructures by using a wide range of photosensitive materials^24–27^. Compared with the traditional micro/nanofabrication technologies, femtosecond laser microfabrication has the distinct advantages of high precision, material-independent versatility in processing, and space-selective fabrication^28^. Specifically, the ultrashort pulse of femtosecond laser significantly suppresses the thermal effects, which ensures high-quality fabrication and further improvement of the spatial resolution^29^. Furthermore, the capability of multiphoton absorption due to its extremely high peak intensity enables the direct 3D processing in transparent materials^30–33^. Combining these techniques will hold great potential in creating a 3D microchip with 3D integrated microstructures. Tickunas et al. reported a glass-polymer micromechanical sensor, which could investigate the elastic properties of polymeric microstructures fabricated by hybrid fabrication^34^. A passive LOC particle separator was successfully fabricated to separate polystyrene spheres in an aqueous mixture using hybrid subtractive-additive-welding microfabrication^35^. Bragheri et al. reported a sorter fabricated by a hybrid 3D processing technique^36^. Meanwhile, Paie et al. used the same hybrid fabrication to process a microfluidic 3D hydrodynamic focusing device^37^. In our previous works^38^, cell counters were realized by our originally developed technique, which consisted of hybrid subtractive (femtosecond laser-assisted wet etching of glass (FLAE)) and additive (two-photon polymerization (TPP) of polymer) 3D processing. All of these works have accelerated the progress of the FLM technology for integrated microchips. However, these works are about the integration of high-precision 2D–3D microcomponents in single-layer microchannels.

Currently, model systems drive biological research by recapitulating body processes and functions from the molecular level to the whole organism level. However, the lack of technology that recapitulates “multi organ interaction” prevents us from testing how each organ interacts with one another to affect the functions of other organs at the cellular levels. Thus, it is important to increase the degree of integration for multifunctional applications. One effective strategy is to adopt multilayered microfluidic systems. Because of the difficulty in realizing uniform etching for different layers, it remains challenging to fabricate multilayered microchannels based on glass. In addition, the high-precision integration of 3D microcomponents into multilayered microfluidics in a designable manner is challenging for the current FLM.

In this paper, we develop an optimized hybrid processing technology to realize “all-in-one” multilayered microchips, which demonstrates the fabrication of 3D multilayered glass microfluidic channels integrated with different 3D polymer microstructures in each layer. Glass was selected for microchip fabrication because it is more stable, has better mechanical strength than paper or PMMA and has good optical transmittance for optical analyses. Multilayered microfluidic channels with good stability and high surface smoothness were facilitated in a single glass chip by FLAE. FLAE can directly create multilayered microfluidic channels embedded in glass without the procedures of photolithography and stacking and bonding of substrates with a single operation. To ensure the uniformity of the entire 3D multilayer glass microchips, two strategies were newly proposed. One strategy was to optimize the FLAE laser powers for different layers to ensure the consistency of laser power deposited at each layer. The other strategy was to prepare the control layers (nonlaser exposed regions) at the upper ends of the longitudinal channels to simultaneously complete the etching of all layers. The height of the “control layer” and the laser parameters were systemically investigated and optimized. Solvents with different dyes were used to verify that each layer was isolated from one another. For the polymer structure integration by TPP in 3D multilayered microchannels, three important parameters (prebaking time, laser power and developing time) were quantitatively optimized to secure the high quality at every layer. Since both glass and polymer SU-8 are transparent materials, both have great potential in biochip application, particularly for observation, detection, and analysis by optical means. The use of this hybrid “all-in-one” femtosecond laser process to fabricate 3D integrated multilayered microfluidic chips can expand the applications of biochips for multi-cellular culture systems with 3D configurations.

## Results and discussion

### Quantitative investigation of FLAE-laser power to fabricate 3D multilayered microfluidic channels

**Fig. 1:**
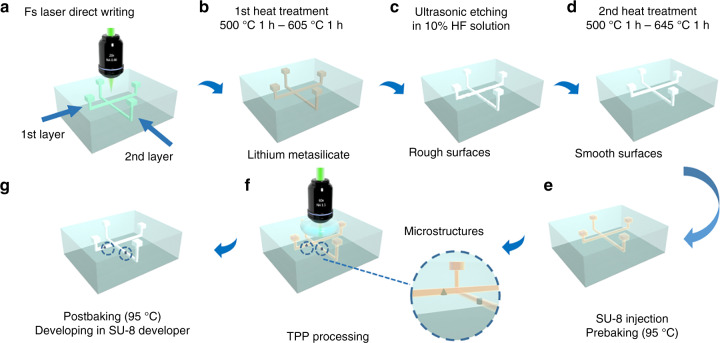
**Schematic illustration of the fabrication procedure for 3D multilayered microfluidic chips integrated with polymer microcomponents by hybrid “all-in-one” femtosecond laser processing**. **a**–**d** FLAE of a photosensitive glass to fabricate 3D multilayered glass microfluidic channels, which involves **a** femtosecond laser direct writing with the “control layer” strategy, **b** first heat treatment, **c** HF etching, and **d** second heat treatment to improve the surface smoothness. **e**, **f** Integration of high-precision polymer 3D microstructures in 3D embedded multilayered microchannels at different depths by TPP with optimized parameters. **e** SU-8 injection into the fabricated multilayered microchannels and prebaking with different times, which depend on the channel depth. **f** Direct femtosecond laser writing for TPP with different laser powers for different depths. **g** Developing to remove the unsolidified liquid resin after postbaking. All schematics in this figure are illustrated based on the two-layered microfluidic chip, where each channel is crossed with a right angle

Figure 1 schematically illustrates the fabrication procedure for a 3D multilayered microfluidic chip integrated with polymer microcomponents by hybrid “all-in-one” femtosecond laser processing. Since the polymer microstructures are created in the embedded multilayered glass microchannels, it involves two main steps. The first step is applying FLAE to a photosensitive glass to fabricate the 3D glass microchannel. The second step is the integration of 3D polymer microstructures into 3D glass microchannels by TPP. One of the largest advantages of the “all-in-one” technology is that both steps can be performed using the same femtosecond laser system, which simplifies the procedure and reduces the fabrication cost. Furthermore, the combination of subtractive (FLAE) and additive (TPP) 3D processing exploits specific advantages of each process while diminishing the drawbacks to create a much more complex configuration of microchips with higher functionalities. [Fig. S1].

To realize 3D multilayered microfluidic channels, a key issue is to ensure the uniformity of the shape of each layer. The high uniformity of the shape may be achieved by controlling two factors: the laser power and the etching time. Since optical loss was caused by multiphoton absorption and optical aberration of fs laser during the propagation in the glass substrate, higher laser power should be used to compensate for the energy loss at the deeper layers. This compensation will make each layer of the microchannel have the same modification to obtain the same etch effect during the corrosion process. In this experiment, a line-to-line scanning mode with a pitch of 2 μm is adopted to fabricate four layers of 3D embedded microchannels, where the top layer is embedded 200 μm below the glass surface, and the underlying layers have a 200-μm spacing from one another. Thus, the laser power density^39^ is optimized to 13 ± 1, 13.8 ± 1.3, 15.08 ± 1 and 16.9 ± 1.56 TW/*cm*^2^ for the 1st, 2nd, 3rd, and 4th layer microchannels, respectively Fig. 1a. The FLAE laser power density was measured after M4 Lens (Fig. S1). Each channel is arranged in parallel crosses Fig. S2a.

After the laser scanning, the microchannels are subjected to the first annealing. Due to the free-electron generation by the nonlinear multiphoton absorption of femtosecond laser, Ag^+^ in the irradiated region can capture the electrons to be reduced to Ag atoms. Then, the sample is put in a programmable furnace for the 1st heating treatment Fig. 1b. The temperature is increased to 500 °C at a rate of 5 °C/min, held constant for 1 h, increased to 605 °C at a rate of 3 °C/min and held constant for another hour. Ag atoms diffuse to form Ag clusters at 500 °C, which can act as nuclei to grow the crystalline phase of lithium metasilicate around them at 605 °C. Then, the annealed glass microchip was put in an aqueous solution of 10% hydrofluoric acid (HF) for ultrasonic etching to selectively remove the laser exposed regions Fig. 1c.

### Optimal design and fabrication of 3D multilayered microchannels using the “control layer” strategy

**Fig. 2:**
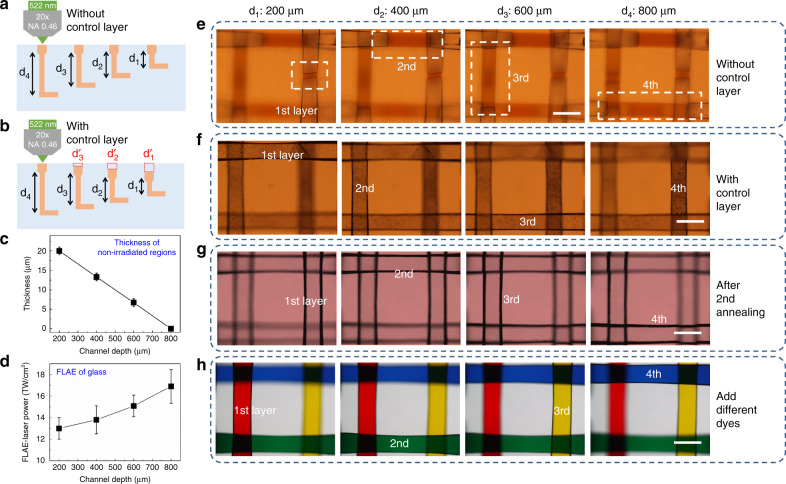
**Fabrication of a 3D multilayered microchannel structure with optimized parameters**. Schematic illustration of the fabrication for four-layered microchannels without (**a**) and with (**b**) control layers. **c** Thickness of the control layer (nonirradiated regions) in FLAE as a function of the channel depths to form at different layers (1–4 layers). The thickness is ~15, ~10, ~5, and ~0 μm for the 1st, 2nd, 3rd, and 4th layer channels, respectively. **d** Relationship of the FLAE laser power and channel depth. The laser powers required for the 1st, 2nd, 3rd, and 4th layer channels are 13±1, 13.8±1.3, 15.08±1 and 16.9±1.56 TW/cm^2^, respectively. Generally, a deeper channel requires more laser power. **e** Optical microscopy image for the four-layered microchips after HF etching without a control layer. A deeper depth corresponds to longer unetched areas. **f** Optical microscopy image for the 4-layered microchips after HF etching with the control layer. All four layers can be simultaneously completely etched. **g** Optical microscopy image for the 4-layered microchips with high smoothness after the second annealing. **h** Four-layered channels arranged in parallel crosses at depths of 200, 400, 600, and 800 μm by FLAE and filled with red, green, yellow, and blue dye solvents. The four solvents do not mix, which verifies that each layer is well isolated. Scale bars: 200 μm

The laser irradiation of photosensitive glass produces a much higher HF etching rate (typically 30–50 times) in the irradiated areas (the crystalline phase of lithium metasilicate) with respect to unexposed areas^40^. Since the HF etching always begins from the surface of the glass and progresses toward deeper regions, the top layer will suffer a longer etching period than the bottom layer. This issue worsens for fabricating multilayered microchannels with lengths up to a few millimeters and can seriously affect the uniformity of the multilayered microchannels. Therefore, for multilayered microchannels, if all layers are modified at the same irradiation condition of femtosecond laser, they cannot be simultaneously completely etched. As shown in Fig. 2a, e, although the first layer is almost etched away, some irradiated area remains unetched for the second layer. Similarly, the third and fourth layers have the longer and longest unetched areas, respectively. A deeper layer has longer unetched areas (Fig. S3). If the etching time is extended to ensure the complete etching of the fourth layer channel, the first to third microchannels will be overetched, which will seriously affect the overall uniformity of the multilayered microchannels. To solve this problem, a processing strategy called “control layer” is proposed. As mentioned, the etching rate in irradiated areas is 30–50 times higher than that in nonlaser irradiated areas. Therefore, we reserve some unexposed areas near the glass surface for the first to third layers $d_1^\prime$,$d_2^\prime$ and $d_3^\prime$ in Fig. 2b to balance the etching time. For example, the fourth and third layers of the channel have longitudinal channels with lengths of 800 and 600 μm from the upper surface of the glass, respectively. Therefore, the length difference of the longitudinal channels between these two layers is 200 μm, which corresponds to the layer spacing. In the experiment, the contrast ratio in the etching selectivity between the laser exposed and unexposed regions is set to 40, which implies that the etching rate in HF solution for the exposed regions is 40 times higher than that in the unexposed regions. To simultaneously complete the etching of all layers, we prepare an ~5-μm-long unexposed area, which is determined by dividing the spacing length between the third and fourth layers by the etching selectivity (200 μm/40), as the control layer at the two upper ends (glass surface) of the longitudinal channels for the third layer as shown in Fig. 2b. Similarly, compared to the fourth layer, the length difference for second and first layers is 400 and 600 μm, respectively. Then, the length of the control layers at the two upper ends of the second and first layers are designed to be ~10 and ~15 μm, respectively. Figure 2f clearly shows that all four layers with control layers can be simultaneously completely etched. All figures were simultaneously taken.

After HF etching, the microchannels have poor surface smoothness (Fig. 1c and S4). Since these microchannels will be used as a platform to integrate high-precision 3D polymer devices, the high surface smoothness is highly demanded. Then, the microchannels are subjected to the second annealing at an optimized temperature of 645 °C (higher than the 570 °C temperature in the previous work^41^, Fig. 1d). We have confirmed that an annealing temperature above 680 °C results in severe deformation of the microchannel. Using these improved protocols, a highly smooth channel was produced, as shown in Fig. 2g.

To verify that each layer of microchannels is isolated from one another, we injected different colors of dyes into each channel. After filling the microchannels with different dye solvents, we found that the four solvents did not mix together and verified that each layer was completely isolated Fig. 2h. Furthermore, we realized the two- and three-layered structures using the same method [Figs. S2b and c]. For the two-layer structure, each channel was crossed with a right angle, whereas for the three-layer structure, each channel was crossed at its center at an angle of 60°. Figures S5 and S6 show the optical microscope images for two types of multilayered microchannels before and after the dye solvents filled them.

### Quantitative investigation of the key experimental parameters to integrate polymer microstructures into true 3D multilayered microchips

Because of the optimized FLAE processing and good programmability of the laser fabrication facility, high-quality 3D multilayered glass microchannels can be fabricated by a single laser operation without additional procedures, such as stacking and bonding processes. Furthermore, to integrate uniform polymer microstructures in these microchannels, the TPP parameters must be optimized. Since the microchannels are embedded in the glass, the femtosecond laser beam must propagate through the glass to reach the polymer in the microchannels. Thus, the conditions for TPP are different from those on the surface. Our previous work shows that the prebaking time, laser power and developing time are three key parameters for the TPP fabrication.

In this experiment, a commercial epoxy-based negative-type resin SU-8 (2075, MicroChem) is first injected into the 3D multilayered microchannels for TPP fabrication. The resin features high transmittance for light from visible to near-infrared wavelengths, low volume shrinkage during polymerization, good mechanical properties, and high thermal stability (degradation temperature ~380 °C), which makes it a good candidate for the creation of microcomponents in microfluidics.

**Fig. 3:**
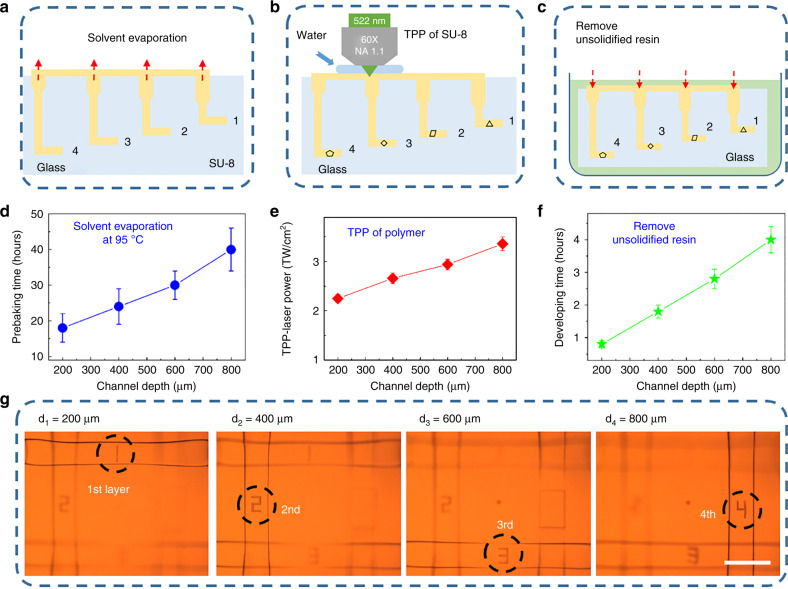
**Quantitative investigation of three key experimental parameters (prebaking time, TPP-laser power and developing time) to integrate polymer microstructures into true 3D multilayered microchips**. **a**–**c** Schematic illustrations of the solvent evaporation, TPP of SU-8 and developing. **d** Relationship of the prebaking time and channel depth. For the deeper channel, longer prebaking time is required to totally vaporize the solvent in the SU-8 resin. The optimized prebaking times were 18 ± 4, 24 ± 5, 30 ± 4, and 40 ± 6 h for the one-, two-, three-, and four-layer microchips, respectively. **e** The TPP laser powers required for the formation of the 1st-, 2nd-, 3rd-, and 4th-layer channels are 2.52±0.08, 2.66±0.11, 2.94±0.11 and 3.36±0.14 TW/cm^2^, respectively. **f** The required developing time for the one-, two-, three-, and four-layered microchips is 0.8 ± 0.1 h, 1.8 ± 0.2 h, 2.8 ± 0.3 h and 4 ± 0.5 h, respectively. A deeper channel requires more developing time. **g** Multilayer integration of different polymer characters of “1”, “2”, “3”, and “4” in each of the four layers. Scale bars: 200 μm

Before TPP, the sample is put in a hot plate to vaporize the solvent in the resin as shown in Fig. 3a. In general, 1 h of prebaking time is adequately long to remove the solvent in SU-8 on the glass surface. However, in the 3D multilayered microchannels, the solvent evaporation proceeds more slowly due to the spatially embedded structures, so the prebaking time must be significantly increased with the increase in channel lengths [Fig. S7]. Consequently, the prebaking times of 18 ± 4 h, 24 ± 5 h, 30 ± 4 h, and 40 ± 6 h are used for the 1st, 2nd, 3rd, and 4th layer channels, respectively (Fig. 3d). Since the prebaking time of each layer is different, attention must be paid to the time when we inject SU-8 to the channels. First, we evenly poured SU-8 into the 4th layer channel. After 10 h of prebaking, SU-8 was poured into the 3rd layer channel. Then, after six more hours of prebaking, SU-8 was poured into the 2nd layer channel. Finally, after 6 h of prebaking, the resin was poured into the 1st layer channel.

In addition to the prebaking time, the laser power is quantified, as shown in Fig. 3e. The commonly used oil-immersion lens cannot be employed for in-channel TPP because its working distance is typically ~220 μm, which is shorter than the depth of the multilayered microchannels. Therefore, we used a water-immersion lens (×60 Olympus) because it combined the long working distance (1.5 mm) with an adequate numerical aperture (NA = 1.1) as shown in Fig. 3b. For traditional TPP fabrication on the glass surface, optical reflection occurs at the air/SU-8 or oil/SU-8 interface. Additionally, a small optical loss occurs during the propagation in the polymer material. Meanwhile, there are three interfaces (water/SU-8, SU-8/glass, and glass/SU-8) for TPP in the embedded multilayered microchannels, which generate more optical reflection. Then, to compensate the power loss due to optical reflection, higher laser powers are used for the deeper layers. Thus, the TPP laser power densities of 2.52 ± 0.08, 2.66 ± 0.11, 2.94 ± 0.11 and 3.36 ± 0.14 TW/*cm*^2^ were adopted for the 1st-, 2nd-, 3rd-, and 4th-layer channels, respectively. In general, the laser polarization affects the additive and subtractive manufacturing^42^. However, in our experiment, the fabricated microchannels and integrated polymer microcomponents are on the order of ~200 and ~20 µm, respectively. The polarization has little effect on the overall scale change, which is difficult to observe under an optical microscope, especially in the enclosed microchips. In the future, when we fabricate nanoscale devices, we will carefully investigate the effect of polarization.

After the TPP integration, the sample was postbaked at 95 °C for 10 min and developed in the SU-8 developer to remove the unsolidified liquid resin Fig. 3c. The developing time for a commercial SU-8 developer is ~0.5 ± 0.2 h. Similarly, the removal speed of the unsolidified liquid resin in the 3D multilayered microchannels is slower due to the much smaller interaction area of the developer and polymer; hence, the deeper microchannels require more developing time [Fig. S7]. Then, the developing times are determined to be 0.8 ± 0.1 h, 1.8 ± 0.2 h, 2.8 ± 0.3 h, and 4 ± 0.5 h for the 1st-, 2nd-, 3rd-, and 4th-layer channels, respectively (Fig. 3f).

As a proof-of-concept for demonstration, high-precision polymer microcharacters “1”, “2”, “3”, and “4” were successfully integrated at different layers of the multilayered microchips based on the optimized experimental parameters for both FLAE and in-channel TPP as shown in Fig. 3g.

### Eight-layered microfluidic chip integrated with polymer microstructures by hybrid “all-in-one” femtosecond laser processing

**Fig. 4:**
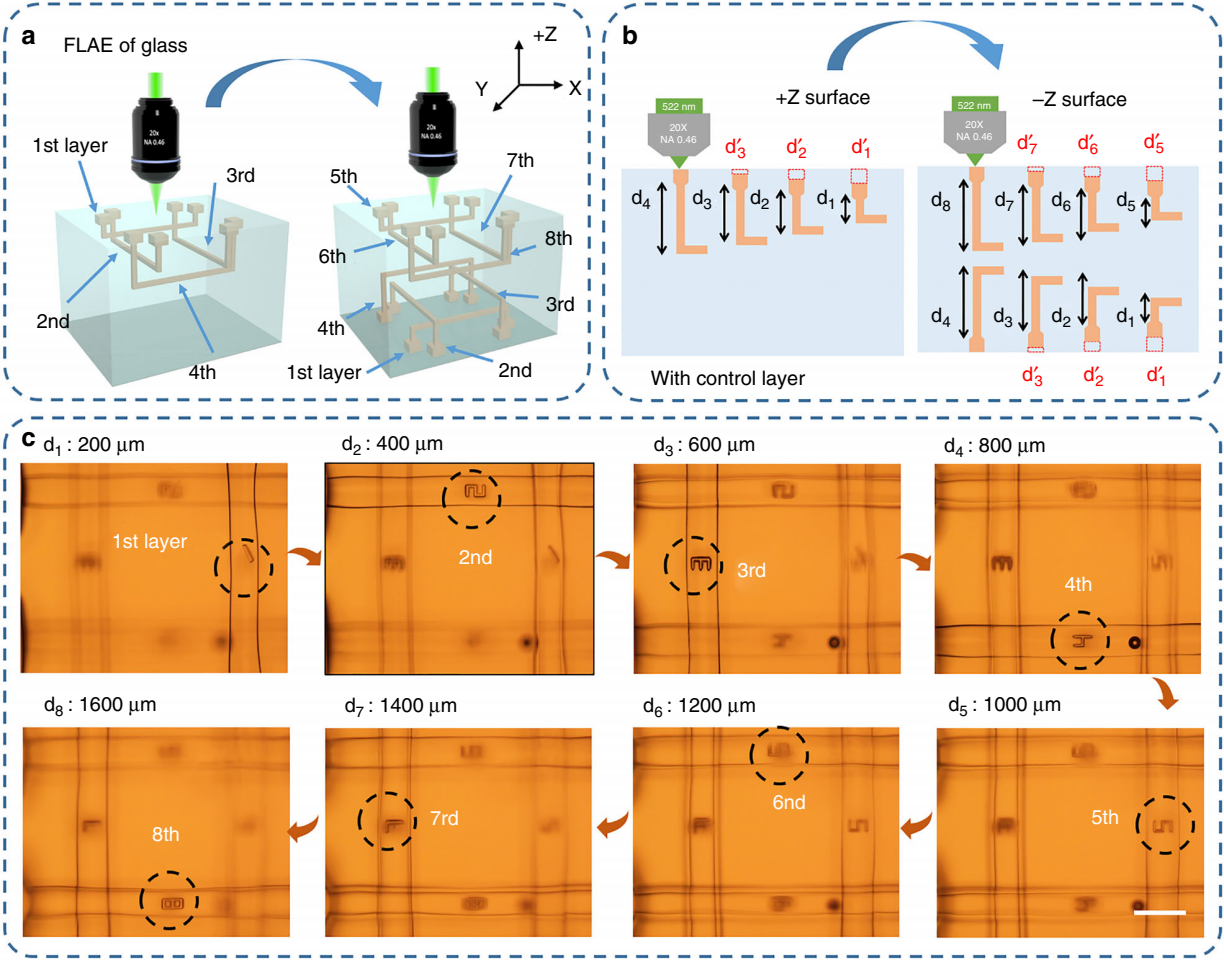
**True 3D eight-layered microchip fabricated by hybrid “all-in-one” femtosecond laser processing**. **a** and **b** Schematic illustrations of the fabrication process of eight-layered glass microchannels by combing FLAE of glass and TPP. First, four-layered 3D glass microchannels were fabricated by FLAE on the front side of the glass (+Z surface) with optimized parameters. Then, we turned over the sample to continue fabricating other four-layered microchannels on the opposite side (−Z surface) with the identical processing parameters and symmetric scanning strategy. **c** Integration of different polymer characters of “1”, “2”, “3”, “4”, “5”, “6”, “7”, and “8” in each of the eight layers. Scale bars: 200 μm

To further demonstrate the unique capability of this hybrid technique for realizing true 3D microchips toward future requirements of miniaturization and high integration, an eight-layered microfluidic chip integrated with polymer microstructures was fabricated, as shown in Fig. 4. First, four layers of 3D glass microchannels were fabricated by FLAE from the front side of the glass (as shown in the left (+Z surface) of Fig. 4a, b) using the described optimized parameters. Then, we turned over the top and bottom of the substrate and continued to process four layers of glass microchannels from the opposite surface (from the −Z surface in Fig. 4) with the same processing parameters and symmetric scanning schemes. The control layer has been prepared prior to processing the 1st, 2nd, 3rd and 5th, 6th, 7th layers of microchannels as described in the previous section $d_1^\prime$, $d_2^\prime$, $d_3^\prime$ and $d_5^\prime$, $d_6^\prime$, $d_7^\prime$ in Fig. 4b. Thus, after annealing and HF etching, an eight-layered glass microchannel arranged with a double-side configuration and a highly smooth surface was successfully fabricated [Figs. S8 and S9]. Finally, each microchannel was filled with polymer for the subsequent TPP integration [Fig. S10]. After successive processes of prebaking, laser direct writing, postbaking, and development with the optimized conditions, high-precision polymer microcharacters “1”, “2”, “3”, “4”, “5”, “6”, “7”, and “8” were integrated into each layer of the eight-layered microfluidic channels, as shown in Fig. 4c. In the TPP integration process, we first fabricated the microcharacters “1”, “2”, “3”, and “4” from the +Z surface; then, we turned over the microchip to continue the integration of microcharacters 5”, “6”, “7”, and “8” in other layers from the −Z surface. Ultimately, the eight-layered microchips comprising 3D embedded glass microchannels and high-precision polymer microcharacters were accomplished by hybrid all-in-one femtosecond laser microfabrication [Fig. 4c]. Distinct features of this technique, such as the true 3D capability, flexibility, and simple procedure of TPP can help to fabricate highly integrated multifunctional microstructures to extend the applications of the biochips.

## Conclusion

In conclusion, a 3D multilayered microfluidic chip integrated with polymer microcomponents was fabricated by an optimized hybrid femtosecond laser process, which is called “all-in-one” femtosecond processing. Compared with the conventional microfabrication techniques (e.g., photolithography), the “all-in-one” technology enables the fabrication of 3D embedded multilayer glass microchannels and the integration of high-precision polymer microstructures by only a single femtosecond laser microprocessing system. This hybrid technique can exploit specific advantages of each process while diminishing the drawbacks. Technically, we have solved the following two crucial problems: (1) The FLAE laser power to fabricate microchannels at different depths was optimized, and the control layer strategy was proposed, both of which are crucial for fabricating the multilayered microchannels with high uniformity. (2) We experimentally demonstrated the feasibility of the integration of polymer microstructures in 3D embedded multilayered microchannels at different depths by quantitatively optimizing three important parameters in TPP. The integration was performed in a single layer of embedded microchannel in the previous work. Since the microstructures are postequipped with a given microfluidic chip, there is a wide range of material selection. In addition to SU-8, some natural photosensitive macromolecules are promising for functional microstructure integration, which can be used to fabricate highly biocompatible microstructures including the cell scaffold, cell grippers and 3D biomimetic environments for microchip functionalization. In the future, the proposed “all-in-one” fabrication of 3D multilayered microfluidic chips with various functional components based on different materials has opened a new avenue for more miniaturization and increase in integration degree of biochips. The goal is to construct diverse 3D scaffolds that can culture specific cells to grow different organs at the desired positions in multilayered 3D microfluidic structures.

## Experimental section

### Femtosecond laser-assisted wet etching

A second-harmonic 522-nm femtosecond laser beam, which was generated from an amplified Yb-doped fiber laser (FCPA μJewel D-400 from IMRA America with a wavelength of 1045 nm, a pulse width of 360 fs, and a repetition rate of 200 kHz), was used for the FLAE step. The femtosecond laser with different powers was used with a ×20 objective lens, which has a numerical aperture of 0.46, to write 3D latent images at different depths in Foturan glass (Schott Glass Corp.), which was made of lithium aluminosilicate doped with Ag and Ce ions. (The laser power depends on the channel depth, see Fig. 2d.) After laser scanning, the Foturan glass was annealed in a programmable furnace to form a crystalline phase of lithium metasilicate. The temperature was controlled in four stages: increasing to 500 °C at a rate of 5 °C min^−1^, being held constant for 1 h, increasing to 605 °C at a rate of 3 °C min^−1^, and finally being held constant for 1 h. Then, the annealed glass microchip was put in an aqueous solution of 10% HF for ultrasonic etching. Due to the much faster etching of the crystallized region in HF, the irradiation areas can be selectively etched away, which results in the formation of microchannels in the glass. However, it is difficult to obtain a smooth surface of the microchannels after the chemical etching. Then, the sample was put in a programmable furnace again for the 2nd heat treatment to produce a smooth surface. The temperature was increased to 500 °C at a 5 °C/min rate, increased to 645 °C at 3 °C/min, and held constant for 1 h. As a result, 3D multilayered microfluidic structures with smooth surfaces were formed in the glass.

### Two-photon polymerization

A commercial epoxy-based negative-type resin SU-8 (2075, MicroChem) was injected into the 3D multilayered microchannels for the TPP integration. The processing equipment is common to FLAE. To achieve a higher fabrication resolution, the objective lens of 20X was replaced by the water-immersion lens (×60 Olympus) with a long working distance (1.5 mm). Before the TPP integration, the sample was put in a hot plate to vaporize the solvent in the resin. The temperature was increased to 95 °C at 5 °C/min, and the prebaking time was changed depending on the channel depth (as shown in Fig. 3d). The TPP laser powers were also changed depending on the channel depth (as shown in Fig. 3e). After the TPP integration, the sample was postbaked at a temperature of 95 °C for 10 min and developed in the SU-8 developer for several hours (depending on the channel length, Fig. 3f) to remove the unsolidified liquid resin. After successive processes of prebaking, laser direct writing, postbaking, and development, the 3D polymer microcharacters were successfully integrated into the multilayered microfluidic chips.

## Supplementary information

Supplementary materials
