# Supplementary material for: Multilayered skyscraper microchips fabricated by hybrid “all-in-one” femtosecond laser processing
Source: Microsyst Nanoeng. 2019 May 6;5:17. doi: 10.1038/s41378-019-0056-3 (PMC6500790; doi:10.1038/s41378-019-0056-3)
Supplement: Supplementary file 1 — Supplementary materials [file 41378_2019_56_MOESM1_ESM.docx]

*Supporting Information*

Multi-layered skyscraper microchips fabricated by hybrid “all-in-one” femtosecond laser processing

*Chaowei Wang,^1^ Liang Yang,^1^ Chenchu Zhang,^3^ Shenglong Rao,^1^ Yulong Wang,^1^ Sizhu Wu,^4^ Jiawen Li,^1^ Yanlei Hu,^1^ Dong Wu,^1*^ Jiaru Chu^1^ and Koji Sugioka^2*^*

1. CAS Key Laboratory of Mechanical Behavior and Design of Materials Department of Precision Machinery and Precision Instrumentation, University of Science and Technology of China, Hefei 230026, China
2. RIKEN Center for Advanced Photonics, 2-1 Hirosawa, Wako, Saitama 351-0198, Japan
3. Institute of Industry and Equipment Technology, Hefei University of Technology, Hefei 230009, China
4. School of Instrument Science and Opto-electronics Engineering, Hefei University of Technology, Hefei 230009, China

*Equally contributed author.

Corresponding author E-mail: [dongwu@ustc.edu.cn](mailto:dongwu@ustc.edu.cn) and ksugioka@riken.jp

**Key words:** Hybrid femtosecond laser processing, multi-layered microchips, true 3D microstructure, control layer strategy, two-photon polymerization

**CONTENTS**

**Supplementary Fig.S1** Schematic image of hybrid “all-in-one” femtosecond laser processing system for FLAE of glass and TPP of polymer.

**Supplementary Fig.S2** The top view of design model for multilayered microchannels.

**Supplementary Fig.S3** Optical microscopy image of the 4-layered microchips after HF etching without control layers.

**Supplementary Fig.S4** Optical microscopy image of 2-layered microchannels after HF etching.

**Supplementary Fig.S5** Optical microscopy images of 2-layered microchannels before and after filling the dye solvents.

**Supplementary Fig.S6** Optical microscopy images of 3-layered microchannels before and after filling the dye solvents.

**Supplementary Fig.S7**. Schematic illustrations of the solvent evaporation and SU-8 developing for 2^nd^ and 3^rd^ layer channels.

**Supplementary Fig.S8** Optical microscopy images of 8-layered microchannels after HF etching

**Supplementary Fig.S9** Optical microscopy images of 8-layered microchannels after the second annealing

**Supplementary Fig.S10** Schematic illustration of fabrication process of 8-layered glass microchannels integrated with different polymer microstructures by combing FLAE of glass and TPP of polymer.


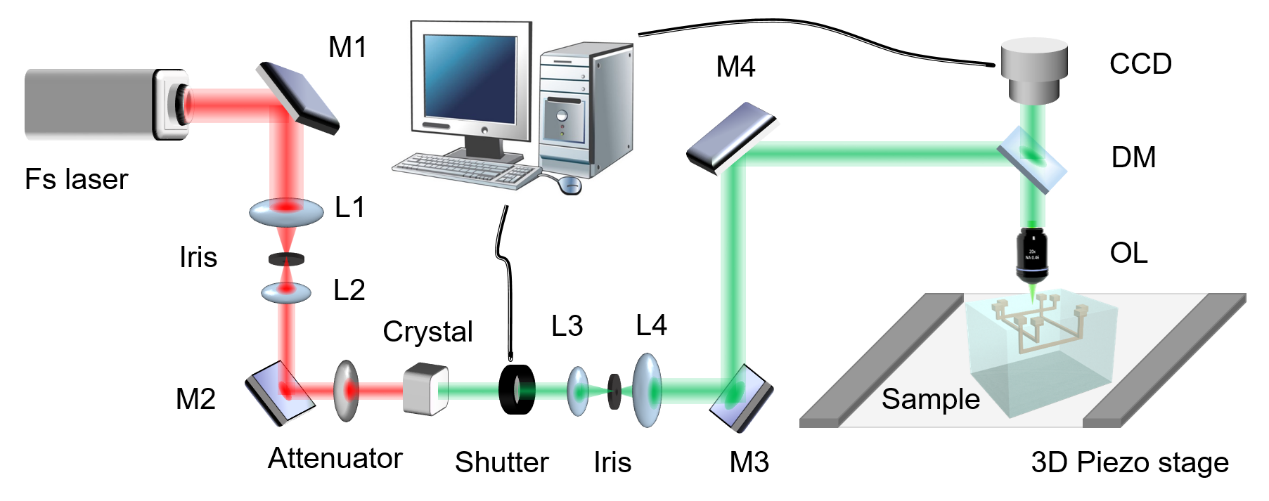


**Figure S1**. Schematic image of hybrid “all-in-one” femtosecond laser processing system for FLAE of glass and TPP of polymer. The M1 to M4 stand for the reflective silver-coated mirrors which have >96% reflectivity from visible ~400 nm to near infrared ~2 μm. Lens 1 (f=200 mm) and Lens 2 (f=50 mm) form a set of beam size reduction system for decreasing the diameter of the laser beam (1045 nm) to increase the efficiency of second harmonic generation (SHG). After passing through the frequency-doubling crystal, the maximum laser power (522 nm) is 50 mW, which is high enough for both FLAE and TPP. The laser power is adjusted with an attenuator. Lens 3 (f=30 mm) and Lens 4 (f=300 mm) form a set of beam expander system for increasing the diameter of 522 nm laser beam to improve the quality of spot center and realize high resolution by fulfilling the numerical aperture of objective lens. For FLAE, a 20× objective (N.A. =0.46) was used, while for polymer microcharacters TPP integration in multilayer microchannels, it was changed to a 60 × water-immersion objective lens. (N.A. = 1.1). The DM stands for dielectric mirrors which is ultra-high reflectivity for certain wavelength (>99% at 522 nm). Most of white light can pass it and reach CCD for in-situ monitoring the sample.


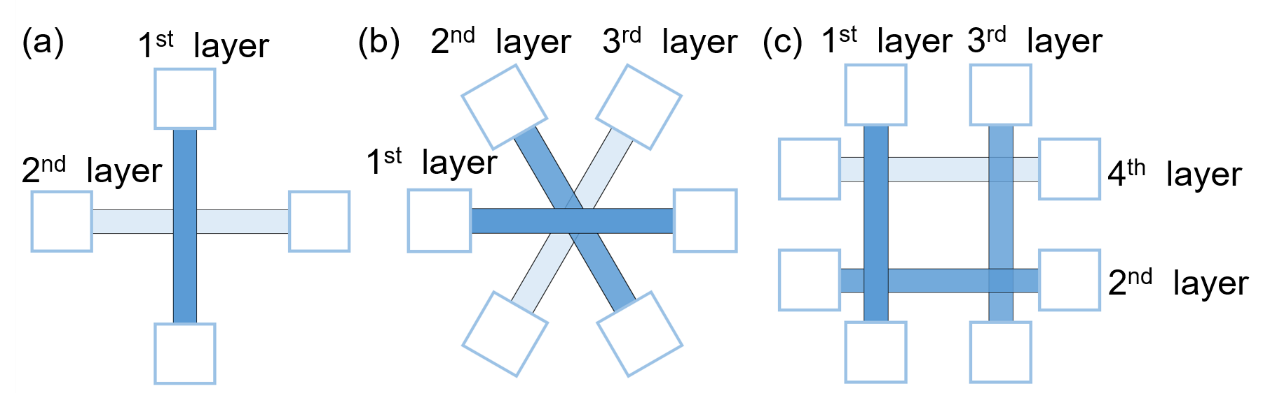


**Figure S2**. The top view of design model for multilayered microchannels. (a) 2-layered structure. Each channel is crossed with a right angle. (b) 3-layered structure. Each channel is crossed at their center at an angle of 60°. (c) 4-layered structure. Each channel is arranged in parallel crosses. All of these multilayered structures are embedded in the glass in which the top layer is embedded 200 μm below the surface and the underlying layers have a 200 μm spacing each other.


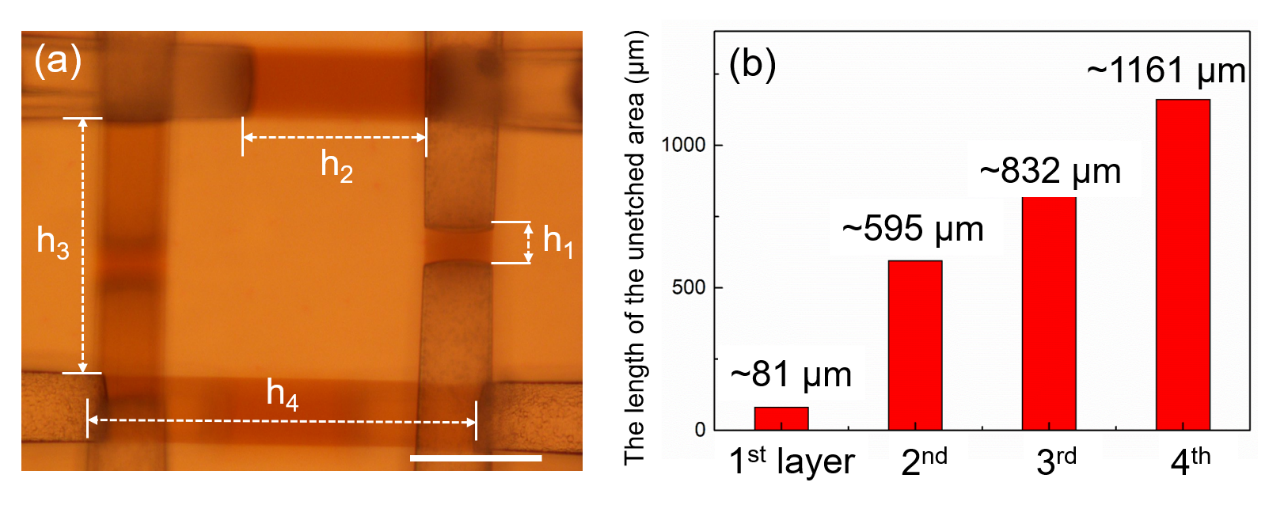


**Figure S3**. (a) Optical microscopy image of the 4-layered microchips after HF etching without control layers. (b) The relationship between the length of the unetched area and the depth of the microchannels. When 81 μm length is left to be etched for the 1^st^ layer, about 595, 832 and 1161 μm lengths are not yet etched for 2^nd^, 3^rd^ and 4^th^ layer. Scale bars: 500μm.


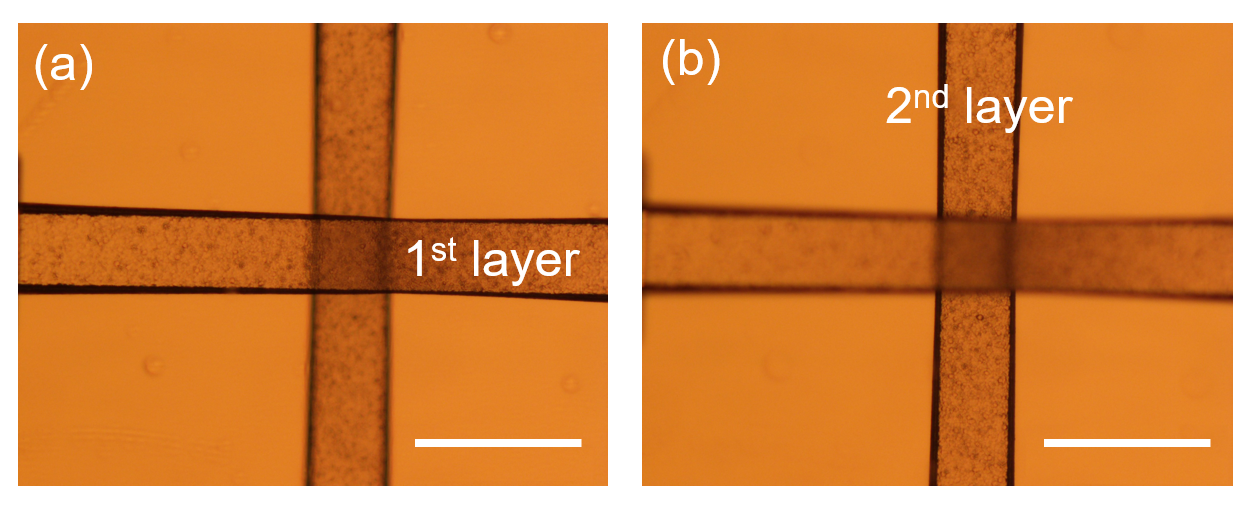


**Figure S4**. Optical microscopy image of 2-layered microchannels after HF etching. It is obviously seen that the microchannels have a poor surface smoothness. Scale bars: 500μm.


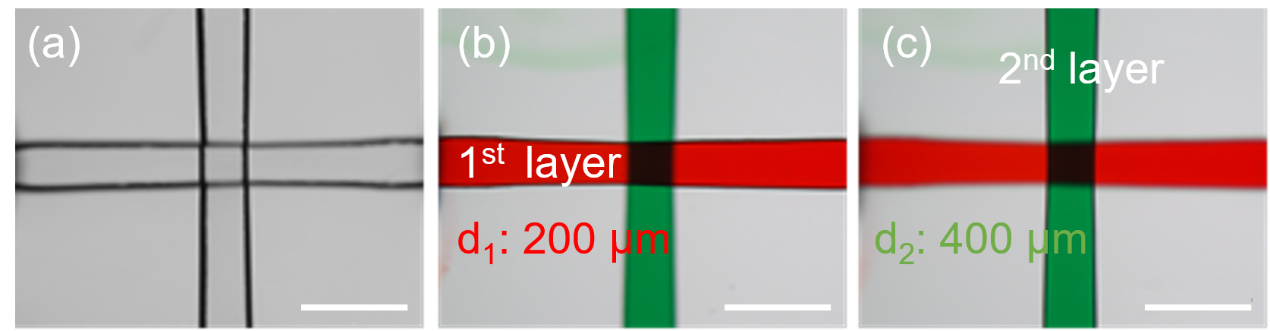


**Figure S5**. Optical microscopy images of 2-layered microchannels before and after filling the dye solvents. (a) With the 2^nd^ annealing, the surface quality of 2-layered microchannels is greatly improved. (b)-(c) 1st and 2nd layers of microchannels formed at depths of 200 and 400 μm are filled with red and green dye solvents, respectively. The focus of images were set at the 1st and the 2nd layers in (b) and (c), respectively. The two solvents do not mix each other, verifying that each layer is isolated. Scale bars: 500μm.


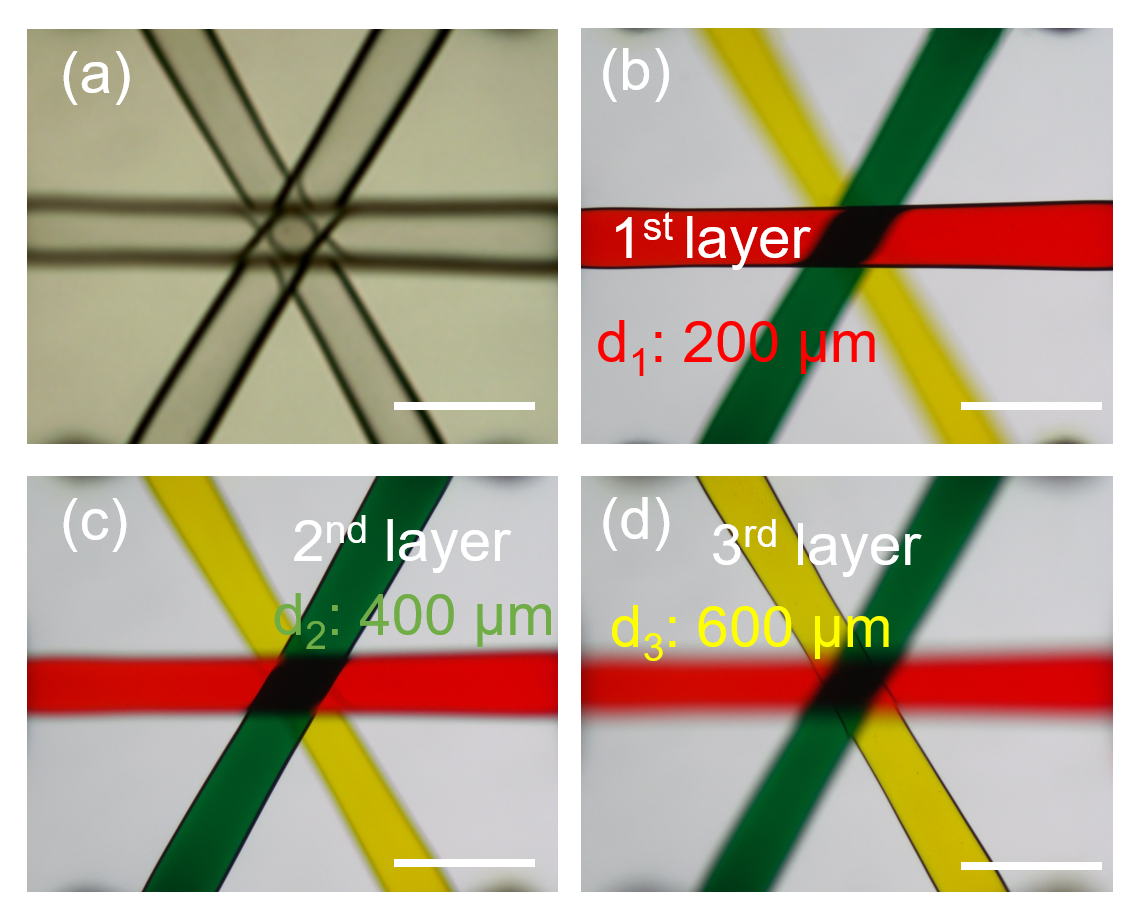


**Figure S6**. Optical microscopy images of 3-layered microchannels before and after filling the dye solvents. (a) With the 2nd annealing, the surface quality of 3-layered microchannels is greatly improved as well. (b)-(d) 1st, 2nd, and 3rd layers of microchannels formed at depths of 200, 400 and 600 μm are filled with red, green and yellow dye solvents, respectively, showing no mixing. Scale bars: 500μm.

Generally, the pre-baking time is about 1 h on the surface, which is sufficiently long to remove the solvent in the resin. However, in 3D embedded channel, evaporation proceeds much slowly so that the pre-baking time needs to be significantly increased with the increasing of channels lengths. In this work, since each layer has the same length parallel to the surface inside the glass, a deeper channel means that the total length of the channels become longer, which takes more time to sufficiently evaporate the solvent in the SU-8 photoresist Then, we simply fit the relationship between the baking time and the length of channels, as followed:

*T_baking time_=0.018*L_total_ -4.4*

Similarly, the developing time for the resin in the channel is much longer than that on the surface. And, in 3D embedded channel, the developing time depends on the channels lengths as well [Fig. R3 (c) and (d)]. We simply fit the relationship between the developing time and the length of channels, as followed:

*T_developing time_=0.0026*L_total_ -2.42*


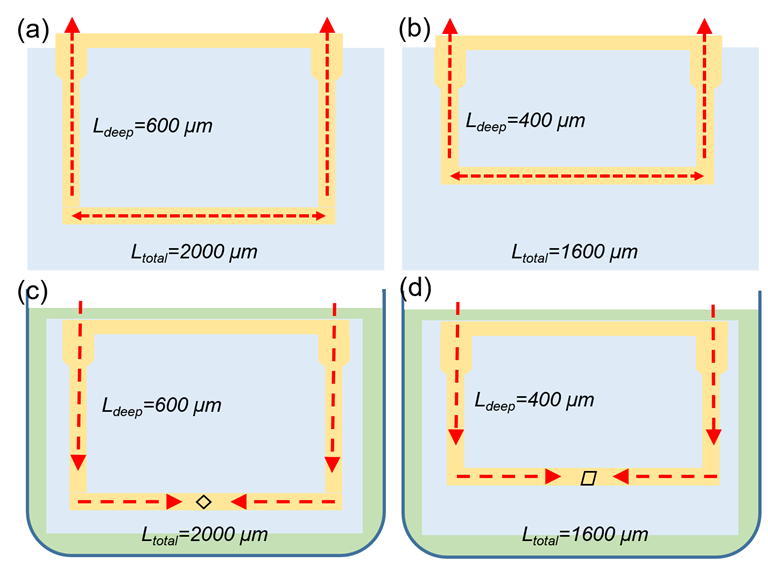


**Figure S7**. Schematic illustrations of the solvent evaporation and SU-8 developing for 2^nd^ and 3^rd^ layer channels. (a) and (c) the solvent evaporation and SU-8 developing for 2^nd^ layer channels. (b) and (d) the solvent evaporation and SU-8 developing for 3^rd^ layer channels. Both the baking and developing times are dependence on the channels lengths. The longer the channels length, the more time for baking and developing completely.


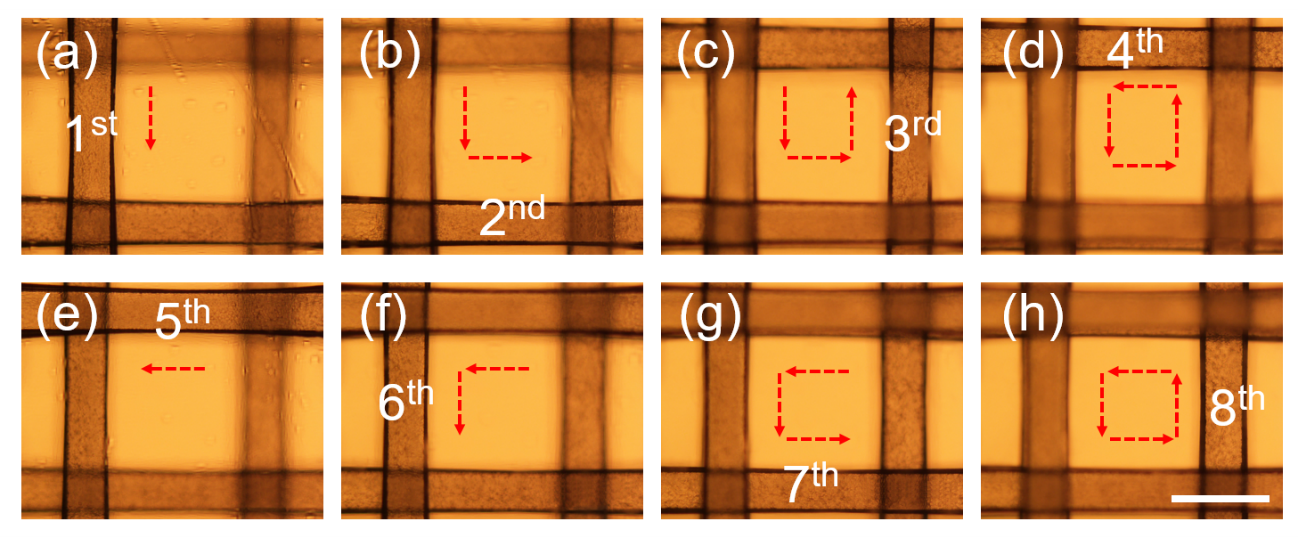


**Figure S8**. Optical microscopy images of 8-layered microchannels after HF etching in which the odd and even number order layers of microchannels are arranged to be perpendicular to each other with a spiral configuration. Scale bars: 500μm.


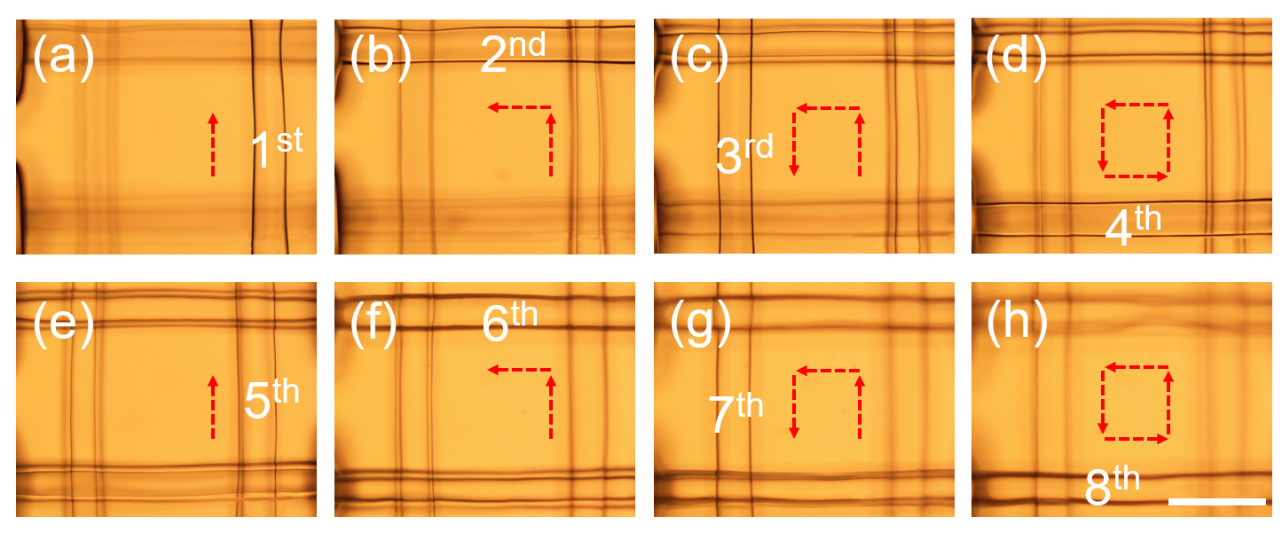


**Figure S9**. Optical microscopy images of 8-layered microchannels after the second annealing, in which the odd and even number order layers of microchannels are arranged to be perpendicular to each other with a spiral configuration. The quality of the surface is improved obviously which is facilitating the subsequent integration of polymer microcomponents. Scale bars: 500μm.


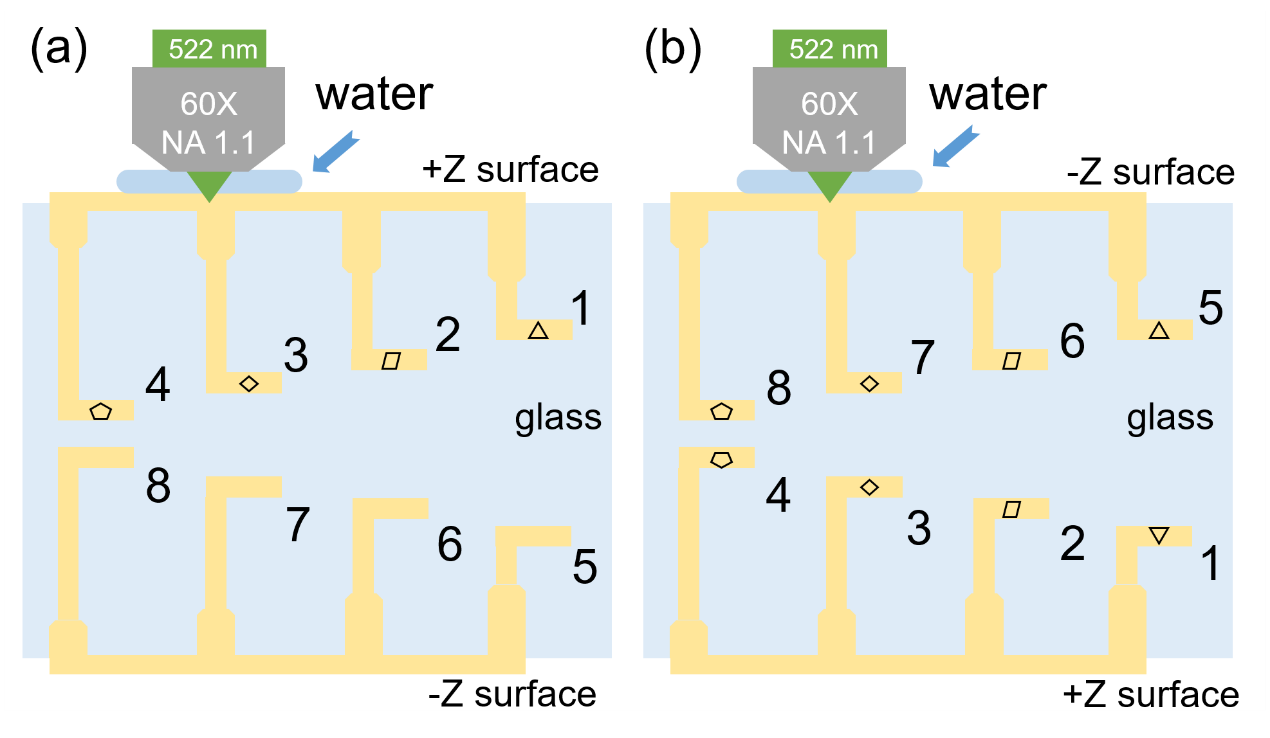


**Figure S10**. Schematic illustration of fabrication process of 8-layered glass microchannels integrated with different polymer microstructures by combing FLAE of glass and TPP of polymer. (a) The microcharacters are first integrated in the 1st to 4th layers from the front side of the microchips (+Z surface) with the optimized laser powers. (b) Then, we turn over the sample to continue integration of the microcharacters in the 5th to 8^th^ layers from the opposite side (-Z surface) with the same processing parameters.
